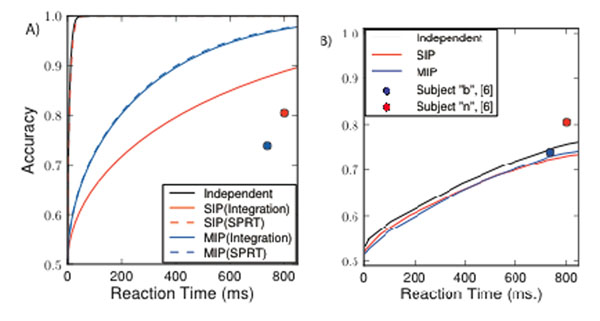# Speed and accuracy in decision making: input correlations and performance

**DOI:** 10.1186/1471-2202-13-S1-P44

**Published:** 2012-07-16

**Authors:** Nicholas Cain, Eric Shea-Brown

**Affiliations:** 1 Applied Mathematics, University of Washington, Seattle, WA 98388, USA

## 

In models of perceptual decision making, evidence for and against different task alternatives is encoded in the firing rates of sensory neurons, and a downstream computation or circuit integrates this evidence over time and makes a decision. What are the consequences of correlations among the sensory neurons for performance in the decision task? We answer this question for three models of decision-making: exact spike integration, the sequential probability ratio test, and a physiologically based model of decision making consisting of 2000 model neurons [1]. Others [2,3] have have previously reported that without correlations, spike integration implements optimal inference via the SPRT [4] by accumulating the log of the likelihood ratio for two alternatives until a threshold is reached. We extend these results to incorporate correlations among the sensory neurons. We compare the performance of each decision making model by computing the accuracy that they produce at a given mean reaction time. Because each decision model receives identically formatted inputs, our approach is to directly compare each model by examining mean reaction time and accuracy together, across the frontier of average performances attainable by a change in decision making threshold.

Our results are as follows. First, we find that weak correlations diminish performance for the spike-integrating model, relative to the performance of an accumulator implementing the SPRT (see Figure 1(A), difference between black, and blue/red curves). Second, using two different models with identical pairwise correlations (SIP and MIP processes [5]), but correlations differing at higher orders , we find that higher-order interactions significantly impact the performance of the spike integrator (see Figure 1(A), difference between, and blue and red solid curves). Finally, we compared the performance curves obtained by the 2000 neuron spiking model, receiving independent, and SIP/MIP correlated inputs. Although this model performs the worst, it is surprisingly insensitive to weak input correlations and compares well with experimental data [6] (Figure 1(B)).

**Figure 1:**